# Genome characterization of prunus maculavirus 1 (PrMcV-1), a novel member of the genus *maculavirus* identified in *prunus* spp.

**DOI:** 10.1007/s00705-025-06346-x

**Published:** 2025-06-27

**Authors:** Raied Abou Kubaa, Ashrafou Ouro-Djobo, Kristian A. Stevens, Olufemi J. Alabi, Maher Al Rwahnih

**Affiliations:** 1 Department of Plant Pathology, University of California, Davis, CA 95616 USA; 2 Foundation Plant Services, University of California, Davis, CA 95616 USA; 3 Department of Plant Pathology & Microbiology, Texas A&M AgriLife Research and Extension Center, Weslaco, TX 78596 USA; 4 Departments of Computer Science and Evolution and Ecology, University of California, Davis, CA 95616 USA; 5 Department of Plant Protection, School of Agriculture, The University of Jordan, Amman, Jordan

**Keywords:** *Prunus* spp., *Maculavirus*, PrMcV-1, HTS

## Abstract

A novel virus with a (+)ssRNA genome was identified in a symptomless peach tree by high-throughput sequencing. The complete RACE-verified genome of the virus, tentatively named "Prunus maculavirus 1" (PrMcV-1; GenBank accession number PV231830), is 6,664 nucleotides (nt) long, excluding the poly(A) tail, and contains two open reading frames (ORFs). ORF1 (nt 64-5895) encodes a large replication-associated polyprotein, which contains conserved domains associated with methyltransferase (Mtr), papain-like protease (Pro), helicase (Hel), and RNA-dependent RNA polymerase (RdRp). ORF2 (nt 5571–6478) partially overlaps ORF1 and encodes the capsid protein (CP). Phylogenetic analysis based on both ORFs placed PrMcV-1 in a distinct clade within the genus *Maculavirus* of the family *Tymoviridae*. Experimental evidence showed that PrMcV-1 is graft-transmissible. This study expands the known host range of maculaviruses to include *Prunus* species.

**Supplementary Information:**

The online version contains supplementary material available at 10.1007/s00705-025-06346-x.

The family *Tymoviridae* consists of isometric, positive-sense, single-stranded RNA viruses that are currently assigned to the genera *Tymovirus*, *Marafivirus*, and *Maculavirus* [1]. Several criteria have been established by the International Committee on Taxonomy of Viruses (ICTV) for genus assignment within this family [2]. These include mechanical or vector transmissibility, cellular localization of their elicited membraned vesicles, and the number and molecular mass of the encoded capsid protein (CP) subunits. In addition to these biological and physicochemical criteria, the three genera can also be distinguished by the number of open reading frames (ORFs) and the presence or absence of a conserved sequence that serves as a subgenomic RNA (sgRNA) promoter, with tymovirus and marafivirus genomes containing a “tymobox” and a “marafibox”, respectively, while maculavirus genomes lack such a sequence. Across the family, the common species demarcation criteria in the genus are genome (< 80%) or CP (< 90%) sequence identity thresholds and serological specificity [2].

During routine screening of prunus germplasm obtained from a public breeding program in 2020, a novel virus was detected in a scion peach plant accession via high-throughput sequencing (HTS) at Foundation Plant Services (FPS), University of California, Davis. The HTS analysis utilized total RNA extracts from bark tissue obtained using a MagMAX Plant RNA Isolation Kit (Thermo Fisher Scientific, Waltham, MA), following the manufacturer’s protocol. The RNA was first depleted of its ribosomal RNA content and then used for complementary DNA (cDNA) library preparation using a TruSeq Stranded Total RNA with Ribo-Zero Plant Kit (Illumina, Inc., San Diego, CA). The cDNA was single-end sequenced on an Illumina NextSeq 500 platform, generating approximately 23.3 million reads, each 75 nucleotides (nt) in length. Bioinformatic analysis of the HTS reads was done as described previously [3], resulting in the assembly of five contigs (308-3,760 nt) representing the nearly complete genome sequence of a novel virus related to members of the genus *Maculavirus*. No other virus-like contigs were found in the sample. To obtain the complete sequence of the viral genome, RT-PCR was performed with specific primers to bridge the gaps between the HTS contigs by Sanger sequencing (Supplementary Table S1). 5′ and 3′ RACE assays were also performed to verify the genome ends, using a SMARTer RACE 5′/3′ Kit (Takara Bio USA, Inc.) according to the manufacturer’s recommended protocols. The entire viral genome sequence was assembled from the HTS, RT-PCR, and RACE sequence fragments using Geneious Prime 2025 (https://www.geneious.com).

The full genome of the novel virus, tentatively named "Prunus maculavirus 1" (PrMcV-1; GenBank accession number PV231830), is 6,664 nucleotides (nt) long, excluding the poly(A) tail. The genome contains two open reading frames (ORFs), a 63-nt 5′ untranslated region (UTR) and a 3′ UTR of 208 nt (Fig. 1A). ORF1 (nt 64 − 5,895) encodes a 216.5-kDa replication-associated polyprotein that contains several conserved domains essential for viral replication, including viral methyltransferase (Mtr, nt 394-1,239), which is potentially involved in mRNA capping, ensuring efficient viral RNA translation and stability, helicase (Hel, nt 2,998-3,693), which putatively facilitates RNA strand separation during replication, and the RNA-dependent RNA polymerase (RdRp, nt 4,306-5,430), which is likely to be essential for viral genome replication and transcription. ORF2 (nt 5,571-6,478) overlaps ORF1 and encodes the capsid protein (CP), which is putatively involved in virion assembly and host interactions [4–6]. Notably, the predicted molecular mass of the CP of PrMcV-1 is in the range expected for maculaviruses (24.5–25 kDa). In contrast, the CPs of tymoviruses are about 20 kDa, and marafiviruses have two CP cistrons (24–25/21 kDa). The PrMcV-1 genome also contains a tymovirus endopeptidase (Peptidase_C21, nt 2,443-2,721), a cysteine protease responsible for cleaving the viral polyprotein at specific sites [7]. Neither a “tymobox” nor a “marafibox” was found in the PrMcV-1 genome.

**Fig. 1:**
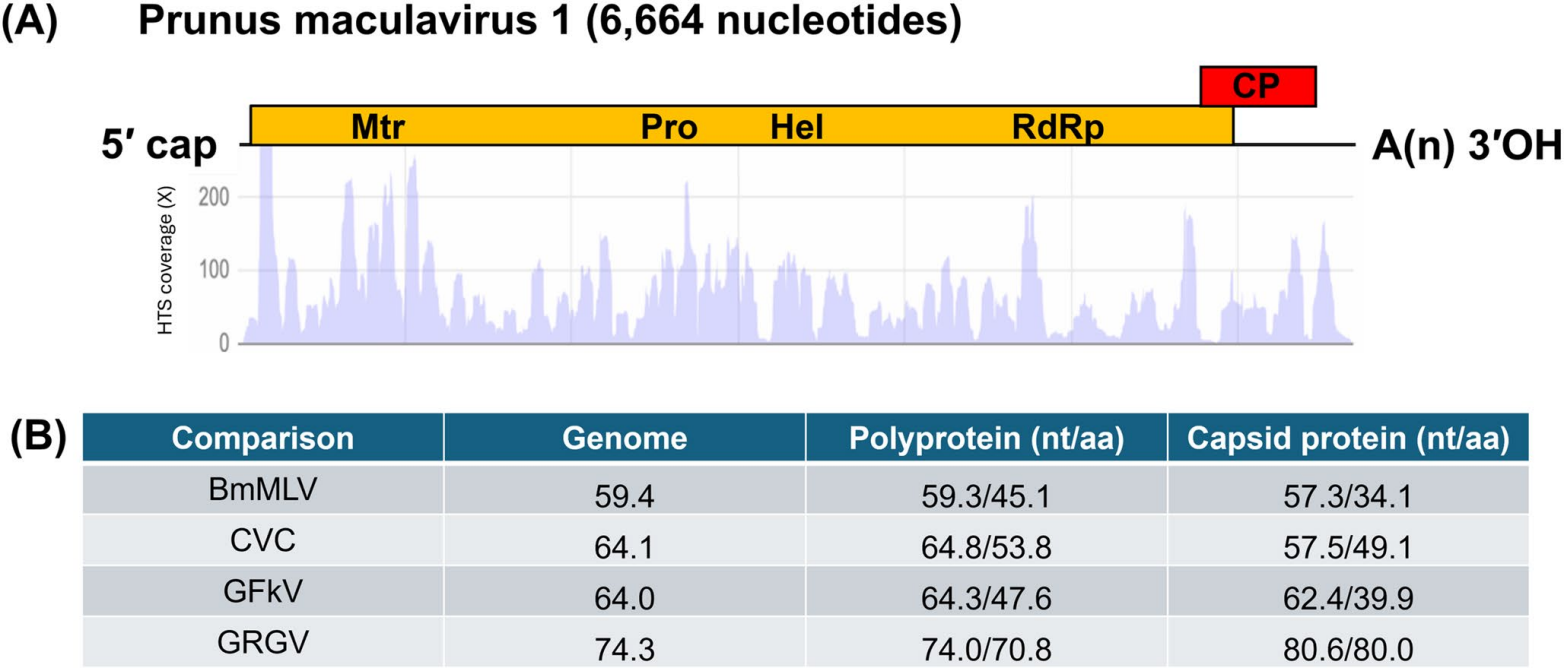
(**A**) Diagram of the genome structure of Prunus maculavirus 1 (PrMcV-1; GenBank accession number PV231830) showing the relative position of the open reading frames (ORFs) and their expression products. ORF1 (nt 64 − 5,895) encodes a replication-associated polyprotein of 216.5 kDa that contains domains for methyltransferase (Mtr), papain-like protease (Pro), helicase (hel), and RNA-dependent RNA polymerase (Pol). ORF2 (nt 5,571-6,478) partially overlaps the replication polyprotein and encodes a capsid protein (CP) of 24.8 kDa. The extent of the genome coverage obtained via high-throughput sequencing (HTS) is also shown. (**B**) Percent (%) pairwise identity between PrMcV-1 and other maculaviruses, including Bombyx mori macula-like virus (BmMLV; NC_015524), citrus virus c (CVC; MN879754), grapevine fleck virus (GFkV; AJ309022), and grapevine red globe virus (GRGV; PP458371). The analysis was performed using the program SDT v1.2 [9]. The sequence-based criteria for demarcating species in the genus *Maculavirus* are (i) overall genome sequence identity less than 80% and (ii) capsid protein sequence identity less than 90% [2].

Maximum-likelihood phylogenetic analysis was performed using amino acid (aa) sequences of (a) the replication polyprotein and (b) the complete CP of representatives of the different taxa of the family *Tymoviridae*, employing the rtREV + G + F model [8] and 1,000 bootstrap replicates. The corresponding aa sequences of potato virus X (PVX; genus *Potexvirus*, family *Alphaflexiviridae*) were included as an outgroup. The analysis based on the replication polyprotein placed PrMcV-1, grapevine red globe virus (GRGV), and citrus virus C (CVC) in a distinct subclade within the genus *Marafivirus* (Fig. 2A), whereas in the analysis based on the CP, all three viruses clustered with grapevine fleck virus (GFkV) and Bombyx mori macula-like latent virus (BmMLV) within the genus *Maculavirus* (Fig. 2B). Members of the genera *Tymovirus* and *Marafivirus* formed separate distinct clades, as expected. According to the *Tymoviridae* Subgroup of the ICTV (https://ictv.global/report_9th/RNApos/Tymoviridae), the genomes of marafiviruses and maculaviruses possess a similar polyprotein ORF; hence, the obtained clustering pattern is not unusual. In pairwise comparisons, the sequence identity of PrMcV-1 to GFkV, CVC, GRGV, and BmMLV based on the complete genome, replication polyprotein, and the capsid protein was found to be below the species demarcation threshold (Fig. 1B). PrMcV-1 was found to share 74.3% nt sequence identity in the complete genome, 74% nt/70.8% aa identity in the polyprotein, and 80.6%nt/80% aa identity in the capsid region with GRGV, its closest relative. Based on the ICTV-established species demarcation threshold criteria of < 80% genome or < 90% CP sequence identity, it can be concluded that PrMcV-1 is a member of a distinct species in the genus *Maculavirus*.

**Fig. 2:**
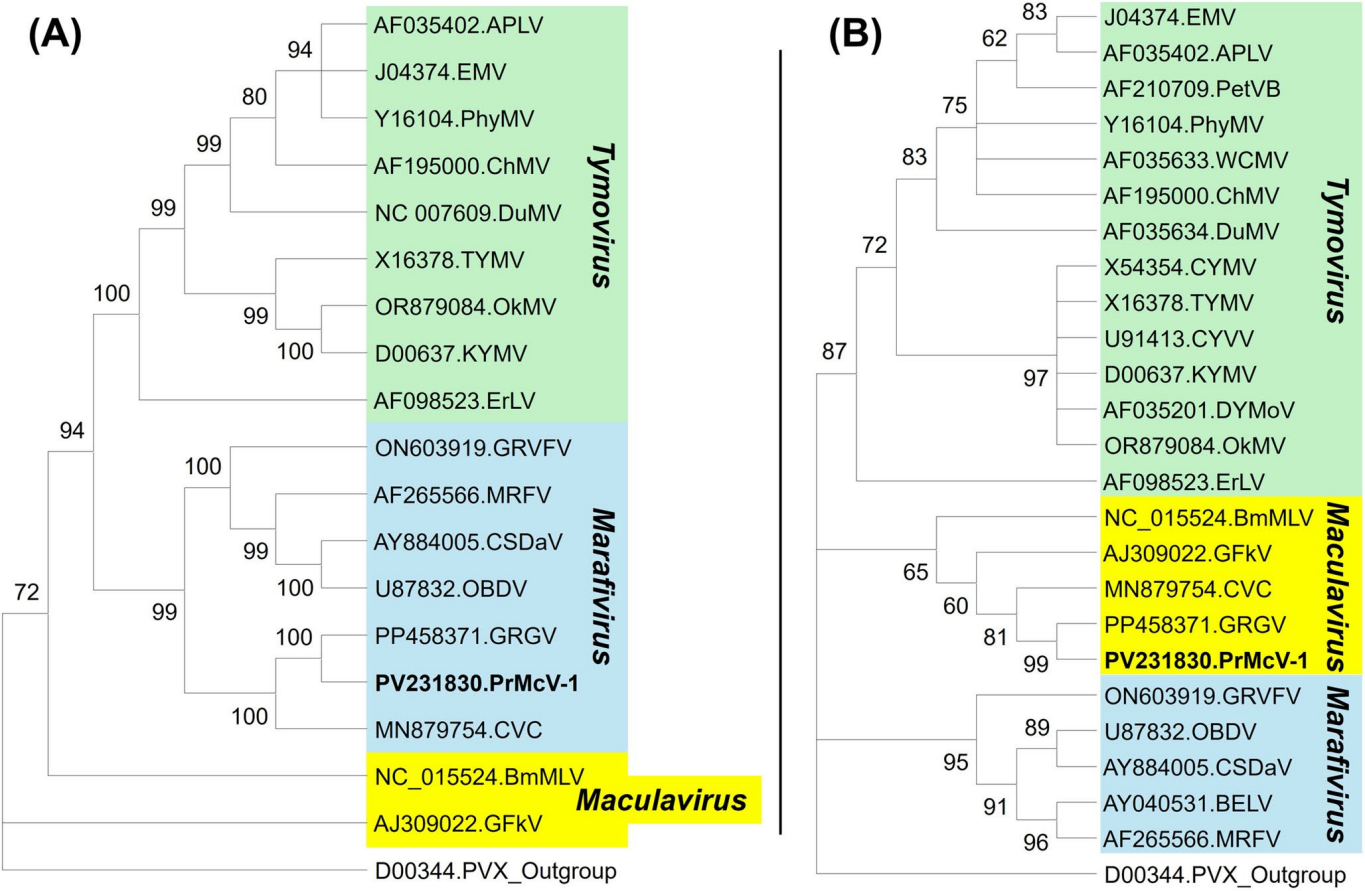
Maximum-likelihood phylogenetic trees (rtREV + G + F model [8]) showing the relationship between the newly characterized Prunus maculavirus 1 (PrMcV-1; GenBank accession number PV231830) and established or tentative members of the different genera of the family *Tymoviridae*. The trees were constructed based on amino acid (aa) sequences of the open reading frame (ORF) 1-encoded replication polyprotein (**A**) and the ORF2-encoded capsid/virion proteins (**B**) of members of the family *Tymoviridae* and corresponding aa sequences of potato virus X (PVX; genus *Potexvirus*, family *Alphaflexiviridae*) as an outgroup. The trees with the highest log likelihood (ORF1 = -36278.61; ORF2 = -7239.04) are shown, and the percentage of replicate trees in which the associated taxa clustered together in the bootstrap test (1,000 replicates) are displayed next to the branches. The analyses included 19 ORF1 and 25 ORF2 amino acid sequences, including those of PrMcV-1, eggplant mosaic virus (EMV), Andean potato latent virus (APLV), petunia vein banding virus (PetVB), wild cucumber mosaic virus (WCMV), chayote mosaic virus (ChMV), physalis mottle virus (PhyMV), Dulcamara mottle virus (DuMV), cacao yellow mosaic virus (CYMV), turnip yellow mosaic virus (TYMV), calopogonium yellow vein virus (CYVV), kennedya yellow mosaic virus (KYMV), desmodium yellow mottle virus (DYMoV), okra mosaic virus (OkMV), erysimum latent virus (ErLV), Bombyx mori macula-like virus (BmMLV), grapevine fleck virus (GFkV), citrus virus c (CVC), grapevine red globe virus (GRGV), grapevine rupestris vein feathering virus (GRVFV), oat blue dwarf virus (OBDV), citrus sudden death-associated virus (CSDaV), Bermuda grass etched-line virus (BELV), maize rayado fino virus (MRFV), and PVX as an outgroup.

To confirm the presence of PrMcV-1 and assess its transmissibility through grafting, two bud chips from the PrMcV-1-infected materials were grafted onto two healthy *Prunus persica* var. Lovell rootstock seedlings. One year after inoculation, leaf petioles and root tip samples were collected and tested by RT-PCR using two pairs of newly designed primers, PrMcV-1_506F (5'-CCTCTTGCACAACGTCTGGA-3')/PrMcV-1_1283R (5'-GTTTTGAAGGTTGTCCCAGGC-3') and PrMcV-1_4609F (5'-AGTACCAACGGTGCTTGGAC-3')/PrMcV-1_5033R (5'-ATTGGTGACTGCGAGGTTGT-3'), followed by Sanger sequencing of the resulting amplicons. The virus was successfully detected in both tissue types, confirming its graft-transmissibility and systemic movement *in planta*, but no symptoms were discernible in the inoculated plants.

Although no symptoms have thus far been associated with PrMcV-1 infection, either naturally or experimentally, further studies are necessary to determine whether the virus contributes to any physiological changes in its host over time, either alone or in concert with other *Prunus*-infecting viruses. In summary, this study represents the first complete genome characterization of a maculavirus naturally infecting *Prunus* spp. PrMcV-1 is phylogenetically related to GRGV (Fig. 2) but should be assigned to a separate species based on the two sequence-based species demarcation criteria within the different genera in the family *Tymoviridae*, as shown in Fig. 1B. Further studies are needed to determine the host range of PrMcV-1, its distribution across production areas, and its possible contributions to disease outcomes, either alone or in coinfections with other *Prunus*-infecting viruses. The species name "*Maculavirus pruni"* is proposed for PrMcV-1.

## Electronic Supplementary Material

Below is the link to the electronic supplementary material

Supplementary Material 1

Supplementary Material 2: Primers used for the amplification of genome gaps between the HTS contigs of prunus maculavirus 1 (PrMcV-1) and for the verification of its genome ends using 5′ and 3′ RACE assays
