# Supplementary material for: Genome characterization of prunus maculavirus 1 (PrMcV-1), a novel member of the genus maculavirus identified in prunus spp
Source: Arch Virol. 2025 Jun 27;170(8):168. doi: 10.1007/s00705-025-06346-x (PMC12204892; doi:10.1007/s00705-025-06346-x)
Supplement: Supplementary file 2 — Supplementary Material 2: Primers used for the amplification of genome gaps between the HTS contigs of prunus maculavirus 1 (PrMcV-1) and for the verification of its genome ends using 5′ and 3′ RACE assays [file 705_2025_6346_MOESM2_ESM.docx]

**Supplementary Table S1** Primers used for the amplification of genome gaps between the HTS contigs of prunus maculavirus 1 (PrMcV-1) and for the verification of its genome ends using 5′ and 3′ RACE assays

| Primer name† | Sequence (5′-3′) | Product size (bp) |
| --- | --- | --- |
| PrMcV-1_A_F | GCCGGTTACATCCGAACCCAA | ~1300 |
| PrMcV-1_A_R | GAGACTGCAACAAGGAGGCAGTC |  |
| PrMcV-1_B_F | TTCACCATCATCCACACCGCA | ~1100 |
| PrMcV-1_B_R | GTAAAGGGAGAAGAGGGAGTTG |  |
| PrMcV-1_C_F | CCTCACCTCTAGACGCACGAC | ~1200 |
| PrMcV-1_C_R | CATTGGTGACTGCGAGGTTGTA |  |
| PrMcV-1_510_F | CCTCTTGCACAACGTCTGGA | ~660 |
| PrMcV-1_1170_R | CGCGAACAGGTTGGTGTAGA |  |
| PrMcV-1_2444_F | CTCGTGACTGCCTCCTTGTT | ~864 |
| PrMcV-1_3308_R | TCTCCTTGAATTGGGTCGCC |  |
| PrMcV-1_4697_F | AGTACCAACGGTGCTTGGAC | ~340 |
| PrMcV-1_5037_R | ATTGGTGACTGCGAGGTTGT |  |
| PrMcV-1 3′GSP | CCTCGGCCCCGCCGGCGCAGTCCGCTCC | ~1500 |
| PrMcV-1 5′NGSP | GAAGAGGACGGCTGAGGGTTGGTGGGC | ~600 |
| PrMcV-1 5′GSP | CTTCAATTCCTCGAAGTTGGGATTGGCC | ~700 |

†F, forward primer; R, reverse primer; GSP, gene-specific primer; NGSP, nested GSP
